# Involvement of LPA_1 _receptor signaling in the reorganization of spinal input through Abeta-fibers in mice with partial sciatic nerve injury

**DOI:** 10.1186/1744-8069-4-46

**Published:** 2008-10-15

**Authors:** Weijiao Xie, Misaki Matsumoto, Jerold Chun, Hiroshi Ueda

**Affiliations:** 1 Division of Molecular Pharmacology and Neuroscience, Nagasaki University Graduate School of Biomedical Sciences, Nagasaki 852-8521, Japan; 2 Department of Molecular Biology, Helen L. Dorris Neuropsychiatric Disease Institute, The Scripps Research Institute, 10550 North Torrey Pines Road, ICND118, La Jolla, CA 92037, USA

## Abstract

Lysophosphatidic acid receptor subtype LPA_1 _is crucial for the initiation of neuropathic pain and underlying changes, such as up-regulation of Ca^2^+ channel α_2_δ-1 subunit in dorsal root ganglia (DRG), up-regulation of PKCγ in the spinal dorsal horn, and demyelination of dorsal root fibers. In the present study, we further examined the involvement of LPA_1 _signaling in the reorganization of Aβ-fiber-mediated spinal transmission, which is presumed to underlie neuropathic allodynia. Following nerve injury, the phosphorylation of extracellular-signal regulated kinase (pERK) by Aβ-fiber stimulation was observed in the superficial layer of spinal dorsal horn, where nociceptive C- or Aδ-fibers are innervated, but not in sham-operated wild-type mice. However, the pERK signals were largely abolished in LPA_1 _receptor knock-out (*Lpar1*^-/-^) mice, further supported by quantitative analyses of pERK-positive cells. These results suggest that LPA_1 _receptor-mediated signaling mechanisms also participate in functional cross-talk between Aβ- and C- or Aδ-fibers.

## Findings

Peripheral nerve injury often accompanies with neuropathic pain, which is characterized by stimulus-independent persistent pain or abnormal sensory perception of pain such as hyperalgesia (exaggerated pain sensations as a result of exposure to mildly noxious stimuli) and allodynia (pain perception on exposure to innocuous tactile stimuli) [1,2]. The mechanisms of allodynia in particular have been long speculated to involve abnormal spinal input through the sprouted myelinated Aβ-fibers that normally conduct innocuous tactile stimuli [3,4]. Moreover, the functional cross-talk between the damaged peripheral sensory fibers causing abnormal spinal input is also postulated to function in neuropathic allodynia [5]. Phosphorylation of ERK (pERK) has been reported as a specific marker for activated cells responding to nociceptive stimuli [6,7] and recent studies using immunohistochemical analysis of pERK provided evidence for spinal reorganization through Aβ-fibers in neuropathic pain models [8,9]. Most recently we have reported that the acute nerve injury caused Aβ-fiber-induced pERK signals within the superficial region of spinal cord dorsal horn, where nociceptive C- or Aδ-fibers are innervated [8]. In addition, it was found that such novel Aβ-fiber-mediated pERK signals in injured mice were blocked by NMDA receptor antagonists that specifically block the nociceptive behaviors induced by C- or Aδ-fiber stimulation. This suggests a pharmacological switch in Aβ-fiber-mediated spinal neurotransmission in injured mice, considering the result that Aβ-fiber stimulation-induced paw withdrawal behaviors were specifically blocked by AMPA/kainate receptor antagonist in naïve mice [8]. These results suggest further that functional reorganization of Aβ-fiber input to the spinal neurons innervated by nociceptive C- or Aδ-fibers may underlie mechanisms for neuropathic allodynia.

We previously demonstrated that LPA_1 _receptor signaling is involved in the initiation of peripheral nerve injury-induced neuropathic pain [2,5,10]. The nerve injury-induced neuropathic pain, demyelination and underlying molecular events were attenuated or abolished in LPA_1 _receptor-knock out (*Lpar1*^-/-^) mice. Most recently we also characterized neuropathic pain by a novel electrical stimuli-induced paw withdrawal (EPW) test using a Neurometer^®^. In this test, there was a significant decrease in sensory perception threshold of Aδ- or Aβ-fiber stimulation in mice with partial sciatic nerve injury [8], and this type of allodynia was abolished in *Lpar1*^-/- ^mice [11]. From the fact that the basal nociceptive thresholds in behavioral tests were not affected in *Lpar1*^-/- ^mice [10,11], it is evident that LPA_1 _receptor signaling works only after occurrence of the nerve-injury. This view was supported by the recent finding that nerve injury-induced neuropathic pain is associated with the *de novo *synthesis of LPA, which is produced by a conversion of lysophosphatidylcholine (LPC) through autotaxin (ATX) or lysophospholipase D (lysoPLD) [11,12]. In the present study, we further examined the involvement of LPA_1 _receptor signaling in the spinal reorganization through Aβ-fiber after the peripheral nerve injury.

Male mutant mice lacking the LPA_1 _gene (*Lpar1*^-/-^)[13] and their sibling wild-type mice from the same genetic background (weighing 20–24 g) were used. All procedures were approved by the Nagasaki University Animal Care Committee and complied with the recommendations of the International Association for the Study of Pain [14]. The partial sciatic nerve ligation injury was carried out according to methods described previously [8,11]. On day 7 after the sham or nerve injury operation, significant thermal hyperalgesia and mechanical allodynia were observed in wild-type mice, which were mostly abolished in *Lpar1*^-/- ^mice [10]. The procedures for Aβ-fiber specific electrical stimulation were performed as described previously [8]. Briefly, the electrodes (Neurotron Inc., Baltimore, MD) were fastened with tape to the operated right planter surface and instep of deeply anesthetized mice. After 10 min, transcutaneous nerve stimuli of 2000 Hz with the current intensity of 1000 μA was applied using a Neurometer^® ^CPT/C (Neurotron Inc.) for 1 min. The control treatment was performed without electrical stimulation. Two min after electrical stimulation, mice were immediately perfused with ice-cold PBS, followed by cold 4% paraformaldehyde solution. The spinal cord (L4–5) was removed and cut on a cryostat at a thickness of 30 μm for pERK1/2 (pERK)-immunostaining. The sections were incubated at 4°C overnight with primary antibody (anti-phospho-p44/42 MAP kinase, 1:500, Cell Signaling Technology, MA), followed by incubation with the biotinylated anti-rabbit IgG (1:500, Vector, CA). The immunoreactivity was amplified with ABC kit (Vector, CA) and visualized by incubation with a solution containing 0.02% 3,3'-diaminobenzidine tetrahydrochloride (DAB; Dojindo, Japan). The immunoreactive cells showing S/N ratio of 3.0 or more and a diameter of > 5 μm were counted as pERK-positive neurons, as described previously [8]. The intensity in the gracile fasciculus regions of white matter was considered as background activity. pERK-positive neurons in the superficial laminae (I-II) of dorsal horn from five sections of each mouse were counted. Statistical comparison was performed using one-way ANOVA with Tukey-Kramer multiple comparison post-hoc analysis. The criterion of significance was established at P < 0.05. All results are expressed as means ± S.E.M. from 4–6 separate mice.

**Figure 1:**
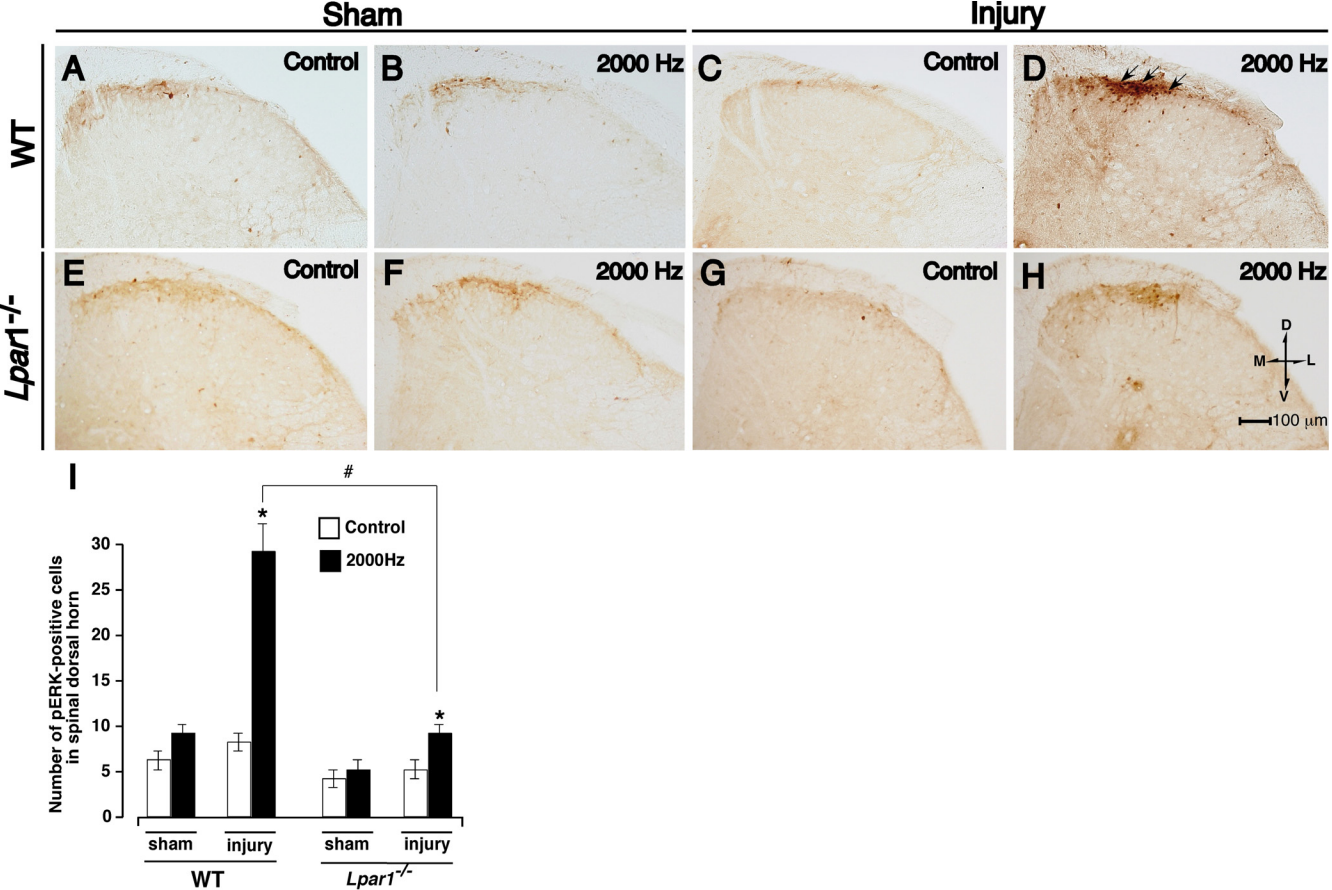
**Lack of Aβ-fiber stimulation-induced ERK activation in *Lpar1*^-/- ^mice after nerve injury.** (A-H) Representative pictures of pERK signals in the ipsilateral spinal dorsal horn after the Aβ-fiber stimulation (2000 Hz) to the right hind paw (see Methods). Arrows in (D) indicate Aβ-fiber stimuli-specific pERK signals observed in wild-type nerve-injured mice. M: medial, L: lateral, D: dorsal, V: ventral. (I) Number of pERK-positive cells per section in ipsilateral dorsal horn. *:*p *< 0.05 *vs*. sham, #:*p *< 0.05 *vs*. wild-type (WT). Data represent the means ± S.E.M. from experiments using 4–6 mice.

In sham-operated wild-type mice, no significant pERK signals were observed by the control treatment without electrical stimulation or by transcutaneous nerve stimuli for Aβ-fiber (2000 Hz, 1000 μA) in the L4–5 spinal dorsal horn (Fig. 1A, B). Although the nerve-injury alone did not induce pERK-signals (Fig, 1C), the Aβ-fiber-stimulation to the paw of nerve-injured mice induced pERK-positive signals in the ipsilateral superficial dorsal horn (laminae I-II), but not in the deeper regions of dorsal horn (lamina III-V) (Fig. 1D), as previously reported [8]. In the sham-operated *Lpar1*^-/- ^mice, on the other hand, neither control treatment nor Aβ-fiber-stimulation induced any pERK signals (Fig. 1E, F). Although the nerve-injury alone also failed to induce pERK signals in *Lpar1*^-/- ^mice, the Aβ-fiber-stimulation-induced pERK signals were largely abolished in nerve-injured *Lpar1*^-/- ^mice (Fig. 1G, H). The number of pERK-positive cells in spinal dorsal horn was also increased in the nerve-injured wild-type mice after the Aβ-fiber stimuli, and this increase was significantly suppressed in *Lpar1*^-/- ^mice (Fig. 1I).

In the present study, we demonstrated that Aβ-fiber-mediated abnormal spinal input after the nerve injury was significantly suppressed in *Lpar1*^-/- ^mice. These data suggest that the nerve injury-induced spinal reorganization through Aβ-fiber is caused by LPA_1 _receptor-mediated signaling. Extracellular signal-regulated kinases (ERKs), major subfamilies of mitogen-activated protein kinases (MAPKs), are phosphorylated following membrane depolarization and Ca^2+ ^influx [15]. As the noxious stimulation rapidly activates ERK in superficial dorsal horn neurons, the pERK expression could be used as a biochemical marker of activated neurons [6]. Interestingly, we previously found that the pERK expression was specific for nociceptive C-fiber- and Aδ-fiber perception through substance P-NK1 receptor- and glutamate-NMDA receptor-dependent spinal transmission, using the transcutaneous electrical nerve stimulations to the hind paw using the Neurometer^® ^[8]. Few ERK activation was observed after innocuous Aβ-fiber-stimulation in sham-operated mice, which is mediated through AMPA/kainate receptor spinal transmission [8]. As previously discussed, the lack of pERK signals after Aβ-fiber-stimulation may be attributable to the insufficient Ca^2+ ^influx for ERK activation in spinal neurons, because AMPA/kainate receptors have lower Ca^2+ ^permeability than NMDA receptors [16]. From the findings that Aβ-fibers are normally innervated to the neurons in lamina III-IV [17], and Aβ-fiber stimulation-induced abnormal pain was blocked by NK1 or NMDA receptor antagonist, but not by AMPA/kainate antagonist [8], it is suggested that Aβ-fiber stimulation may cause a stimulation of nociceptive pain pathway through a functional cross-talk. We speculate that the direct contact among different modalities of fibers and sprouted fibers following LPA_1_-mediated demyelination may underlie the functional cross-talk [2,5]. Alternatively sprouted fibers derived from Aβ-fibers may innervate to the second-order spinal neurons, which are normally innervated by C- or Aδ-fibers [3,18].

In conclusion, LPA_1 _receptor-mediated signaling mechanisms contribute to spinal neuronal reorganization through Aβ-fiber and could contribute to mechanisms underlying neuropathic allodynia.

## Authors' contributions

All authors contributed equally to the work.

## Competing interests

The authors declare that they have no competing interests.
